# Starving the Teeth

**Published:** 1887-09

**Authors:** 


### STARVING THE TEETH.

"Teeth are just as easily starved to death as the stomach," said a lecturer before a Brooklyn audience the other night. "The fact is that you and your fathers have from generation to generation, been industriouly starving your teeth. In one way it is a blessing to have been born of poor parents. What food the poor give their children is of a variety that goes to make strong bones and teeth. It is the outside of all the grains of all cereal foods, that contain the carbonate and phosphate of lime, and traces of other earthy salts, which nourish the bony tissues and build the frame up. If we do not furnish to the teeth of the young that pabulum they require, they cannot possibly be built up. It is the outside of corn, oats, wheat, barley and the like, or the bran so called, that we sift away and feed to the swine, that the teeth actually require for their proper nourishment. The wisdom of man has proven his folly,shown in every succeeding generations of teeth, which become more and more fragile and weak- These flouring mills are working destruction upon the teeth of every man, woman and child who partakes of their fine bolted flour. They sift out the carbonates and the phosphates of lime, in order that they may provide that fine white flour which is proving a whitened sepulchre to teeth."

"Oatmeal is one of the best foods for supplying the teeth with nourishment. It makes the dentine, cementum and enamel strong, flint-like and able to resist all forms of decay. If you have children, never allow any white bread upon your table. Graham bread is made of whole wheat ground, not bolted, so that the bran, which contains the minute quantities of lime, is present. To make a good, wholesome, nourishing bread, take two bowls of wheatmeal and one bowl of white or bolted flour, and make by the usual process. Nothing is superior to Boston brown bread for bone and tooth building. This is made out of rye meal and corn meal. Baked beans, too, have a considerable supply of these lime salts, and should be on your tables, hot or cold, at least three times a week. In brushing the teeth, always brush up and down, *from* the gum instead of across. Brush away from the gum and on the grinding surfaces of your teeth." —*Brooklyn Eagle.*

